# Spatial navigation under threat: aversive apprehensions improve route retracing in higher versus lower trait anxious individuals

**DOI:** 10.3389/fpsyg.2023.1166594

**Published:** 2023-05-11

**Authors:** Florian Bublatzky, Peter Allen, Martin Riemer

**Affiliations:** ^1^Department of Psychosomatic Medicine and Psychotherapy, Central Institute of Mental Health Mannheim, Medical Faculty Mannheim, Heidelberg University, Mannheim, Germany; ^2^Department of Psychology, University of Koblenz-Landau, Landau, Germany; ^3^Department of Creative Technology, Bournemouth University, Dorset, United Kingdom; ^4^Biological Psychology and Neuroergonomics, Technical University of Berlin, Berlin, Germany

**Keywords:** spatial navigation, route retracing, social learning, threat-of-shock, trait anxiety

## Abstract

Spatial navigation is a basic function for survival, and the ability to retrace a route has direct relevance for avoiding dangerous places. This study investigates the effects of aversive apprehensions on spatial navigation in a virtual urban environment. Healthy participants with varying degrees of trait anxiety performed a route-repetition and a route-retracing task under threatening and safe context conditions. Results reveal an interaction between the effect of threatening/safe environments and trait anxiety: while threat impairs route-retracing in lower-anxious individuals, this navigational skill is boosted in higher-anxious individuals. According to attentional control theory, this finding can be explained by an attentional shift toward information relevant for intuitive coping strategies (i.e., running away), which should be more pronounced in higher-anxious individuals. On a broader scale, our results demonstrate an often-neglected advantage of trait anxiety, namely that it promotes the processing of environmental information relevant for coping strategies and thus prepares the organism for adequate flight responses.

## 1. Introduction

When frightened or under high levels of stress, humans often commit errors that would not have occurred under normal conditions (Eysenck et al., 2007). This is specifically apparent in the domain of spatial navigation (Brown et al., 2020; Goodman et al., 2020). While trying to find our way out of an unfamiliar or allegedly threatening environment, we often fail to recognize informative landmarks and take wrong turns. However, people sometimes also assert that they are even better at finding their way when undergoing acute stress (Cornwell et al., 2012). From a motivational perspective, this seems plausible, because perceived threat can act as additional incentive leading to increased navigational performance (Conrad, 2010).

From clinical studies we know that anxiety disorders (panic, agoraphobia, or generalized anxiety) are correlated with reduced spatial navigation skills (Kallai et al., 2007; Mueller et al., 2009). However, the causal relationship between anxiety and spatial navigation remains unclear. On the one hand, increased anxiety is accompanied by a reduced tendency for spontaneous exploration of unfamiliar environments (Kallai et al., 2007), which ultimately should lead to an underdevelopment of navigational abilities (Newcombe, 2019). On the other hand, increased anxiety in novel environments might be initially caused by insufficient navigational skills and reduced differentiation between spatial contexts (de Voogd et al., 2020).

To elucidate the relationship between anxiety and spatial navigation, an important first step consists of investigating the effects of perceived threat on spatial navigation performance. Using a translational variant of the Morris water maze, in which human participants use distal landmarks to navigate to an invisible target, Cornwell et al. (2012) reported increased performance under threat-of-shock conditions. However, the effects of perceived threat on more naturalistic navigational strategies have rarely been investigated in humans (Walz et al., 2016). In real life, route learning is an important navigational skill that allows repeating a once traveled path (route repetition) and retracing a path to find the way back (route retracing). While route repetition is crucial to relocate significant places, route retracing directly relates to avoidance behaviors and defensive responding to external threats, because it helps us to leave a dangerous environment.

Here we examined the behavioral effects of anticipated shock threat on route repetition and route retracing in a virtual environment, as well as their interaction with trait anxiety (Kállai et al., 2009; Robinson et al., 2013; Grillon et al., 2020). We induced a threatening context by instructing participants that they might receive electric shocks when the sky in the virtual environment has a specific color (i.e., night- or daylight). As outlined above, two hypotheses are possible. Threat-of-shock might distract attention and lead to reduced performance. Alternatively, perceived threat might act as an additional incentive and lead to increased navigation performance, especially for participants with higher levels of trait anxiety. Accordingly, we did not specify a direction for the expected effects of threat-of-shock.

In accordance with the idea that route retracing (in contrast to route repetition) is an intuitive and often adaptive behavior in response to environmental dangers (e.g., to navigate away from threats), we expected more pronounced threat effects for the route retracing task as compared to the route repetition task. Furthermore, due to increased susceptibility and attention toward threatening stimuli (Bar-Haim et al., 2007; Mogg and Bradley, 2016; Bublatzky et al., 2020), the effects of perceived threat should increase with higher levels of trait anxiety.

## 2. Methods

### 2.1. Participants

Forty-eight participants (29 females, 19 males; mean age 24.2 years, ranging from 19 to 46, SD = 5.0) were recruited through public advertisements at the Universities of Landau, Mannheim, and Heidelberg and in the local community. Participants received either course credits or monetary compensation.

We included only healthy participants with normal or corrected-to-normal vision. Exclusion criteria were self-report of current acute or chronic medical or psychological problems (e.g., cardiac insufficiency or substance abuse), and medical advice to avoid stressful situations. Questionnaire scores were assessed for depression (BDI-II M = 9.1, ranging from 0 to 29, SD = 7.8; Beck-Depression-Inventory, Hautzinger et al., 1995) and anxiety (state anxiety *M* = 40.7, ranging from 23 to 67, SD = 9.5; trait anxiety *M* = 41.9, ranging from 25 to 69, SD = 10.3; State–Trait-Anxiety Inventory, Spielberger, 1983). We did not exclude data based on *post hoc* inspection of questionnaire scores.

All participants were informed about the general experimental procedure and provided written informed consent prior to their participation. The ethics committee (University of Heidelberg) approved the experimental threat-of-shock protocol, which is in accordance with relevant guidelines and the Declaration of Helsinki.

### 2.2. Navigation tasks

**Figure 1:**
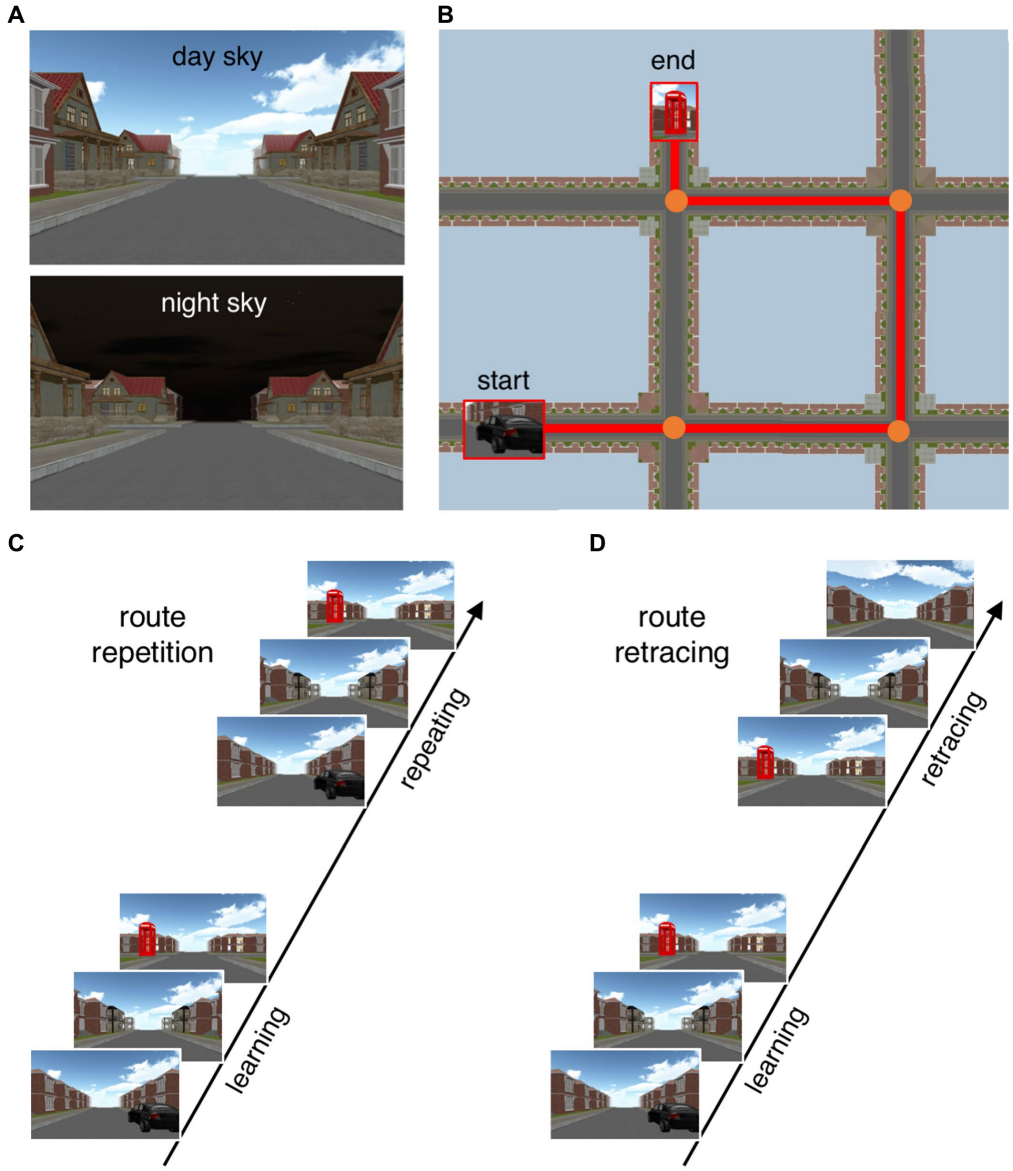
Depiction of the experimental paradigm. **(A)** Navigation tasks were performed with two different sky types (day and night sky) serving as signals for either threat-of-shock or safety. **(B)** Bird’s-eye view illustrating an exemplary route. Start and end points were indicated by a car and a phone booth, respectively. For both navigation tasks, participants were first passively moved along a virtual route including three or four intersections (observational learning phase). **(C)** In the route repetition task, participants then repeated the same route (i.e., from start to end location), indicating the correct direction (left, right, or straight) at each intersection. **(D)** In the route retracing task, participants retraced this route (i.e., from end to start location), indicating the correct direction at each intersection.

Participants performed two navigational tasks (route repetition and route retracing) within a virtual urban environment on a 27-inch computer screen (see Figure 1). The virtual environment consisted of streets and four-way intersections in a residential neighborhood (for a detailed description and download link see Wiener et al., 2020). The buildings at the roadsides were all identical except for the unique corner houses that served as navigational landmarks. The distance between intersections was 105 m and walking speed was 6 m/s. During an initial learning phase, participants were passively transported along a route including three or four intersections (i.e., one-trial observational learning). For the route repetition task, participants were asked to repeat this route in the same direction as during the learning phase. The route retracing task served to assess the participants’ ability to find their way back to the starting point, thus the walking direction was opposite to the learning phase. When participants arrived at an intersection, they could use the arrow keys on a keyboard to indicate the next direction in both tasks. No time limit was set for the decision process on which direction to take. The task was programmed with Unity version 5.2.2f1 (Unity Technologies, 2015).

### 2.3. Procedure and threat induction

Upon arrival in the lab, participants were informed about the general procedures of the experiment, which consisted of a single session, and provided informed consent. Following this, questionnaires were completed and two fake electrodes were attached to the non-dominant inner forearm. Participants were instructed that the electrical stimuli that might occur during the experiment have been rated as ‘maximally unpleasant but not yet painful’. For each task, participants first performed two practice trials to familiarize themselves with the virtual environment, the instructions, and the buttons to be pressed (each trial once with day and night skies; see Figure 1). Data from the practice trials were not recorded.

Participants were randomly assigned to one of three groups. In the night-threat group (10 females, 7 males; mean age 23.1 years, ranging from 19 to 30, SD = 3.6), participants were told that up to three unpleasant electrical shocks might occur when a night sky is present in the virtual environment (threat-of-shock context), but that no shocks will be presented during a day sky (safety context). For participants in the day-threat group (10 females, 6 males; mean age 23.3 years, ranging from 19 to 32, SD = 3.4), threat and safety cues were reversed, that is, the day sky signaled threat and the night sky signalized safety. In the no-threat group (9 females, 6 males; mean age 26.4 years, ranging from 19 to 46, SD = 7.1), participants were assured that no electrical shocks would occur, neither during the day nor the night sky condition. It is important to note that in all groups, no shocks were presented during the entire experiment. This was done to examine the effects of aversive anticipation but not experience of shocks. This threat-of-shock procedure has been shown to reliably provoke persistent psychophysiological defensive responding even across repeated test days without shock application (*cf.* Bradley et al., 2005; Bublatzky et al., 2014, 2022; Schellhaas et al., 2020).

Participants started with either the route repetition or route retracing task (counterbalanced order) and each task was performed six times in a row. Four routes containing three intersections and two additional routes containing four intersections were presented, the latter in order to increase task difficulty. The routes were alternately presented with either a day sky or a night sky. In order to avoid carry-over learning effects, each route was presented only once per task and participant. Across participants, each route was used equally often with a day and night sky. To ensure the comparability of the environments with different sky types, the luminance of the streets and buildings was kept constant. At the end of the experiment, day and night sky environments were rated regarding valence and arousal using the Self-Assessment-Manikin (SAM; Bradley and Lang, 1994) and regarding perceived threat using an 11-point Likert scale (0–10). Ratings were collected using paper-pencil versions of the scales and regardless of task sequence.

### 2.4. Statistical analysis

Data were analyzed in R version 4.1.2 (R Core Team, 2016). Data and analyses scripts can be found at https://osf.io/3qdf9/?view_only=3cdac6088877468c93462cd33413ea7e. Reaction times deviating more than three standard deviations from the individuals’ mean were excluded from the analyses of reaction times (2.7% of the data). For both task types, navigation performance was defined by the percentage of erroneous responses at the intersections and the mean of reaction times for those choices. Data of participants with errors in half of all trials or more were excluded from analysis of navigational performance (3.1%; one participant from the night-threat group was excluded from both tasks, and one participant from the no-threat group was excluded from the route retracing task).

To examine the valence, arousal, and threat ratings for the night- and the day-sky, we fitted a linear mixed effects model (3 × 2 factorial design) using the R packages lme4 (Bates et al., 2015) and lmerTest (Kuznetsova et al., 2017), including the between-subjects factor group (night-threat vs. day-threat vs. no-threat) and the within-subjects factor sky type (night vs. day), and added a random intercept for subjects. For the three-level factor of group, the no-threat group was defined as the reference level. To test for an interaction with trait anxiety, we added this continuous variable as fixed factor in a separate model and compared the models with and without this additional information using function KRmodcomp of R package pbkrtest (Halekoh and Højsgaard, 2014).

In another step, we tested whether aversive anticipations *per se* had an influence on navigational performance. As this question is independent of the sky type cueing threat, data from the three groups were aggregated^1^ according to the factor threat condition (threat-of-shock vs. safety), which was fed into a 2 × 2 factorial linear mixed effects model, also including the within-subjects factor task type (route repetition vs. route retracing). The influence of trait anxiety was tested by adding a dummy factor coding for lower or higher trait anxiety (based on a median split) as a fixed factor in a separate model and comparing the models with and without this additional information. For all analyses, 95% confidence intervals were reported and effect sizes were reported as *β* estimates of the models.

## 3. Results

### 3.1. Ratings of valence, arousal, and threat

**Figure 2:**
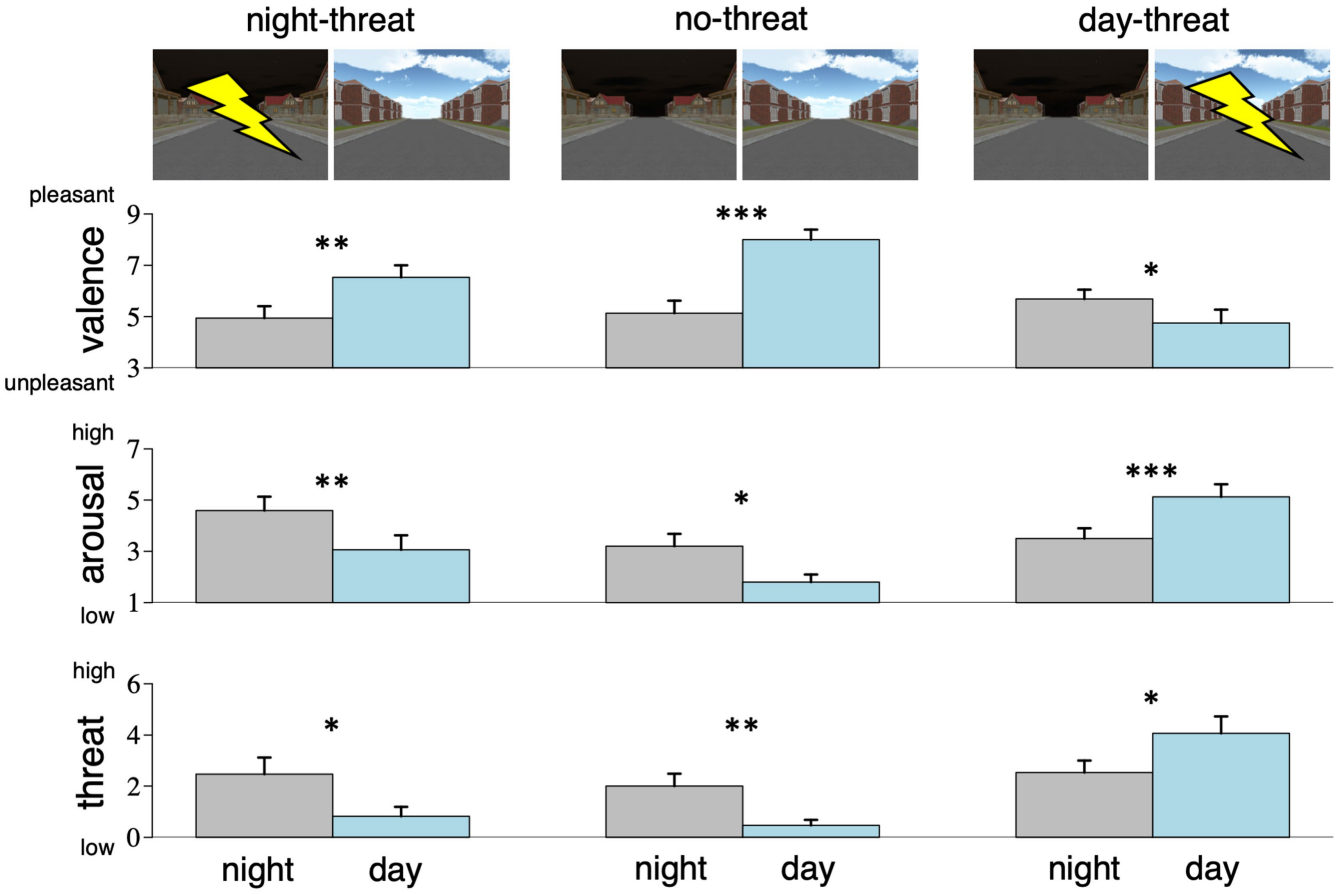
Ratings of valence, arousal, and perceived threat for the night sky (gray) and the day sky (blue), depending on group assignment (threat-of-shock signaled by night or day sky or no-threat). In the no-threat group, the night sky was rated as less pleasant, more arousing, and more threatening than the day sky, and this pattern changed only in the day-threat, but not in the night-threat group. Error bars show standard error across participants. *ps < 0.05; **ps < 0.01, ***ps < 0.001.

Figure 2 illustrates the rating results. With respect to all three measures, the model including *trait anxiety* was not preferred over the base model (valence: *F*_6/65_ = 1.7, *p* = 0.13; arousal: *F*_6/65_ = 0.5, *p* > 0.5; threat: *F*_6/63_ = 1.7, *p* = 0.13).

For all ratings, the base model revealed a significant main effect of *sky type*, indicating that on average the day sky was rated more pleasant (*β* = 2.87, CI_min_ = 1.84, CI_max_ = 3.90, *t*_45_ = 5.4, *p* < 0.001), less arousing (*β* = −1.40, CI_min_ = −2.41, CI_max_ = −0.39, *t*_45_ = −2.7, *p* = 0.010), and less threatening (*β* = −1.53, CI_min_ = −2.72, CI_max_ = −0.34, *t*_44_ = −2.5, *p* = 0.016). We also found significant interactions between *group* and *sky type* which were driven by the difference between the no-threat and the day-threat group (valence: *β* = −3.80, CI_min_ = −5.24, CI_max_ = −2.37, *t*_45_ = −5.1, *p* < 0.001; arousal: *β* = 3.03, CI_min_ = 1.62, CI_max_ = 4.43, *t*_45_ = 4.2, *p* < 0.001; threat: *β* = 3.07, CI_min_ = 1.38, CI_max_ = 4.75, *t*_44_ = 3.5, *p* < 0.001), while a direct comparison between the no-threat and the night-threat groups did not reveal such interactions (valence: *β* = −1.28, CI_min_ = −2.69, CI_max_ = 0.13, *t*_45_ = −1.8, *p* = 0.087; arousal: *β* = −0.13, CI_min_ = −1.51, CI_max_ = 1.26, *t*_45_ = −0.2, *p* > 0.5; threat: *β* = −0.11, CI_min_ = −1.75, CI_max_ = 1.52, *t*_44_ = −0.1, *p* > 0.5). These interaction patterns indicate that day and night sky ratings changed significantly more (relative to the no-threat group) when day was used as a threat cue (*cf.* Figure 2). Visual inspection of Figure 2 and direct comparisons with *t*-tests demonstrate that this difference was due to generally different default ratings of day and night sky in the no-threat group (i.e., without assignment to either threat or safety). Specifically, when none of the sky types was cueing threat-of-shock (i.e., for the no-threat group), the day sky was rated as more pleasant (*t*_14_ = −5.5, *p* < 0.001), less arousing (*t*_14_ = 2.5, *p* = 0.013), and less threatening (*t*_14_ = 3.0, *p* = 0.004) than the night sky. This pattern was reversed only when the day sky was introduced as threat cue (valence: *t*_15_ = 2.0, *p* = 0.030; arousal: *t*_15_ = −4.2, *p* < 0.001; threat: *t*_15_ = −2.5, *p* = 0.012), but not when the night sky was cueing threat (valence: *t*_16_ = −2.9, *p* = 0.005; arousal: *t*_16_ = 2.8, *p* = 0.007; threat: *t*_16_ = 2.5, *p* = 0.012).

### 3.2. Percentage of errors

**Figure 3:**
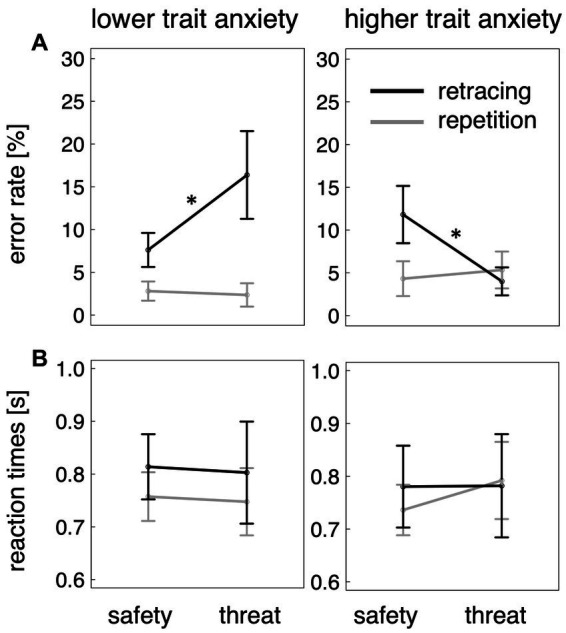
**(A)** Percentage of errors as a function of task type (route repetition vs. route retracing), threat condition (safety vs. threat-of-shock), and lower vs. higher trait anxiety. Only in route retracing did individuals with higher anxiety scores profit from being threatened, while those with lower anxiety scores performed worse when perceiving a threat. **(B)** Reaction times were not significantly different between the conditions. Error bars show standard error across participants. *ps < 0.05.

Results for navigational errors are illustrated in Figure 3A. The base model contained a significant main effect of task type (*β* = 6.83, CI_min_ = 1.59 CI_max_ = 12.07, *t*_114_ = 2.5, *p* = 0.012), indicating that the route retracing task resulted in more errors than the route repetition task. The main effect of threat condition (*β* = 0.03, CI_min_ = −4.85, CI_max_ = 4.88, |*t*_124_| < 0.1, *p* > 0.5) and its interaction with task type (*β* = −0.73, CI_min_ = −7.54, CI_max_ = 6.09, *t*_114_ = −0.2, *p* > 0.5) were not significant.

According to the Kenword-Rogers’ approximation (Halekoh and Højsgaard, 2014), the model including trait anxiety was preferred over the base model (*F*_4/114_ = 4.6, *p* = 0.002). Like the base model, the model including trait anxiety contained a significant main effect of task type (*β* = 15.54, CI_min_ = 8.68, CI_max_ = 22.39, *t*_110_ = 4.4, *p* < 0.001). In addition, it revealed significant interactions between threat condition and task type (*β* = −10.77, CI_min_ = −19.75, CI_max_ = −1.79, *t*_111_ = −2.3, *p* = 0.023), between trait anxiety and task type (*β* = −17.41, CI_min_ = −27.11, CI_max_ = −7.71, *t*_110_ = −3.5, *p* < 0.001), and a significant three-way interaction between threat condition, trait anxiety, and task type (*β* = 19.94, CI_min_ = 7.33, CI_max_ = 32.55, *t*_111_ = 3.0, *p* = 0.003). None of the other effects reached a significant level (all *ps* > 0.39). This indicates, and was confirmed by direct comparisons, that in the route retracing task, individuals with higher trait anxiety make fewer errors during threat-of-shock as compared to safety (*t*_33_ = 2.3, *p* = 0.015), while less anxious individuals tend to commit more errors under threat conditions (*t*_20_ = 1.7, *p* = 0.048). This effect did not occur in the route repetition task (higher trait anxious individuals: *t*_35_ = −0.5, *p* > 0.5; lower trait anxious individuals: *t*_33_ = −0.4, *p* > 0.5).

To further confirm this interpretation of the three-way interaction effect, we performed post-hoc two-tailed correlation analyses, showing that trait anxiety score was negatively correlated with the performance-decreasing effect of threat in the route retracing task (*t*_30_ = −2.8, *p* = 0.009, *r* = −0.45), but not in the route repetition task (*t*_30_ = 0.9, *p* = 0.36, *r* = 0.17). The more anxious participants were, the more they benefited from the aversive context when retracing a route.

### 3.3. Reaction times

Results on reaction times are illustrated in Figure 3B. With respect to the time until participants selected a direction, the base model did not contain any significant effects (task type: *β* = 0.02, CI_min_ = −0.05, CI_max_ = 0.10, *t*_109_ = 0.6, *p* > 0.5; threat condition: *β* = 0.02, CI_min_ = −0.06, CI_max_ = 0.09, *t*_112_ = 0.4, *p* > 0.5; task type × threat condition: *β* = 0.02, CI_min_ = −0.07, CI_max_ = 0.12, *t*_109_ = 0.5, *p* > 0.5). The alternative model including trait anxiety was not preferred over the base model (*F*_4/113_ = 0.1, *p* > 0.5). As the distribution for the reaction time data was positively skewed (skewness of 1.7), we also analyzed logarithmised values. This analysis did not reveal a different pattern of results (all *ps* > 0.5).

The overall non-significant effects on reaction times render it unlikely that the effects on error rates reported in Section 3.2 are due to a speed-accuracy trade-off (i.e., that the commitment of more errors was caused by faster responses during specific conditions).

## 4. Discussion

We investigated the effects of aversive apprehensions on spatial navigation. Spatial navigation is a basic function that helps individuals to relocate significant places and find their way back home. Humans differ greatly regarding their navigational skills, and much research has focused on the effects of aging or medical conditions on spatial navigation (for a review see Wolbers and Hegarty, 2010). Despite this everyday life relevance, few studies have examined the impact of stress-related situations, fear, and anxiety on spatial navigation (Herrero et al., 2006; Conrad, 2010; Cornwell et al., 2012; de Voogd et al., 2020). Our results implicate a dissociation between navigating in threatening and safe environments in high versus low trait anxious individuals. With increasing levels of trait anxiety, participants showed improved navigational performance within a threatening relative to a safe environment, whereas lower-anxious individuals committed more errors under threat conditions. Interestingly, these findings emerged specifically for the route retracing task, which relates to an intuitive avoidance behavior (i.e., running away), but not for the route repetition task, which is more approach-related.

Social communication about potential threats is crucial to organize adaptive behavior. For example, it is enough to be told that a certain neighborhood could be dangerous to raise worries and avoidance of that area; this should be particularly true for individuals prone to anxiety. Such verbal utterances have consistently been shown to cause aversive anticipatory states and trigger psychophysiological reactions that reflect preparatory defensive behavior (e.g., avoidant decision-making, defensive reflex priming; Bradley et al., 2005; Riemer et al., 2015; Bublatzky et al., 2017). We replicate this notion within our experimental manipulation in which the sky of the virtual environment (day and night sky) was instructed as a signal for either threat or safety. As expected, the threatening sky was perceived as more unpleasant, arousing, and threatening compared to the safety sky. With respect to navigation performance, the consequences of verbal threat instructions depended on the level of trait anxiety. Specifically, lower-anxious participants committed more errors in retracing a route within a threatening relative to a safe environment, while higher-anxious individuals navigated better during threat, and thus showed a more adaptive behavioral performance.

While the costs of high trait anxiety and worrying have received much attention (e.g., impaired concentration; Stout et al., 2015), being anxious also has several benefits such as enhanced readiness to avoid dangerous situations and being more sensitive to threat signals (Öhman and Mineka, 2001; Lee et al., 2006). The boundary between advantageous and disadvantageous effects of anxiety and the involved mechanisms are intensively researched, and multiple factors have been identified to modulate cognitive performance (e.g., state−/trait-anxiety, anxiety/stress induction, type of tasks; Byrne and Eysenck, 1995; Bar-Haim et al., 2007; Eysenck et al., 2007; Robinson et al., 2013; Mogg and Bradley, 2016). For instance, according to the attentional control theory (Eysenck et al., 2007), high versus low anxious individuals demonstrate a bias toward processing threat-related stimuli. Given the motivational character of aversive states (e.g., threat-of-shock), highly anxious people should be biased toward stimuli related to intuitive coping behaviors such as avoiding risky decisions (Clark et al., 2012) and running away (i.e., route retracing). Consequently, enhanced motivation to avoid danger might interfere with task performance, unless the task itself requires processing of threat-related stimuli or adaptive coping behaviors (Eysenck et al., 2007). In most studies on cognitive performance, however, performance measures were unrelated to the threat and to potential coping behaviors (e.g., n-back tasks; Vytal et al., 2013), and therefore a reduced performance under threat is often observed (Goodman et al., 2020; Ward et al., 2020). In the case of the route retracing task implemented here, navigation performance is directly related to an adaptive threat-avoidant response (i.e., leaving the dangerous environment), and therefore it is improved. Similar examples for a performance-increasing effect of anxiety have been reported in the field of emotional face perception (Byrne and Eysenck, 1995).

Another finding of the present study is that low trait anxiety was associated with decreased navigation performance under threat conditions, that is, the effect of threat changed its direction for lower (as compared to higher) anxious individuals. This finding cannot be explained in terms of motivational effects of aversive apprehensions for higher anxious individuals, as in this case, one would only expect a less pronounced or absent effect for lower anxious individuals. A possible explanation for this observation consists of the distractive component of being aware of a threat (Eysenck et al., 2007; Derakshan and Eysenck, 2009; Goodman et al., 2020). It is possible that both lower and higher anxious individuals were distracted by the presence of a threat cue, but only for higher anxious individuals did this attention-distracting effect lead to a stronger motivation for effective coping behavior. Lower anxious individuals, in contrast, did not benefit from this motivational boost and the attention-distracting effect resulted in decreased navigation performance.

It is important to note that a non-clinical sample was tested in the current study. While replications in healthy and clinical populations are pending (also with larger group sizes), the suspected mechanisms could be informative for the understanding of maladaptive anxious psychopathology and its etiology. For instance, using a virtual equivalent of a Morris Water Maze task, Mueller et al. (2009) found impaired spatial navigation in children with anxiety disorders (i.e., more heading direction errors and worse accuracy relative to healthy controls). Similarly, disturbed navigational behavior was observed in adults with agoraphobia in an Open Field test (Walz et al., 2016), where participants showed enhanced thigmotaxis, a behavioral tendency to stay close to the sides when exploring open spaces such as a market square. A possible neuronal mechanism for the link between anxiety and spatial navigation performance involves the hippocampal formation within the medial temporal lobe (O’Keefe and Nadel, 1978; Revest et al., 2009). For instance, Jimenez et al. (2018) identified neurons in hippocampal area CA1, which are selectively responsive to locations associated with threats and promote anxiety-related avoidance behavior, and de Voogd et al. (2020) recently demonstrated that a weak representation of spatial contexts in the hippocampus leads to stronger fear generalization from threatening to safe contexts. Thus, spatial navigation tasks provide a useful translational approach to study stress- and anxiety-related pathologies (Schwabe et al., 2008), as threats often occur in the context of specific locations or situations. The present study adds another perspective in showing that the mere anticipation of threat (and not only its experience) modulates behavioral performance with implications for anxious avoidance behavior (e.g., as in agoraphobia).

Several noteworthy aspects, limitations, and future directions should be considered. Regarding spatial navigation, neither environmental threat or safety, nor trait-anxiety modulated performance in the route repetition task. This may relate to conceptual differences of navigational strategies, specifically that route retracing is more avoidance-related (e.g., to find your way back home) compared to the approach-related route repetition task (e.g., to find a significant place again). Moreover, route repetition is cognitively less demanding, as it requires only the memorization of a sequence of navigational decisions (e.g., “left–right-straight”), whereas route retracing additionally requires the inversion of this sequence and swapping of left and right directions. Although some studies suggest that the effects of threat are even more pronounced under conditions of low cognitive load (Vytal et al., 2013), the absence of an effect on the route repetition task could be interpreted as reflecting differences in task difficulty. In future studies, this question can be addressed by increasing the number of decision points along the route (e.g., intersections and/or relevant landmarks).

Another interesting finding relates to the question of whether navigational performance is modulated by the time of day that is associated with threat or safety. According to the preparedness hypothesis (Seligman, 1971; Öhman and Mineka, 2001), the night sky should be more readily understood as threatening relative to the daylight environment, in the same way as it has been shown for angry versus happy facial expressions (Bublatzky et al., 2019). This notion is partly supported by the ratings from the no-threat group, for which neither the day nor night was associated with threat-of-shock. Independent of threat or safety instructions, the night sky was perceived as more unpleasant, arousing, and threatening than the day sky. However, this pattern did not change much for the night-threat group, suggesting that night-threat instructions did match the expectations that a dark environment is more likely dangerous. In contrast, only for the day-threat group, this pattern changed in favor of the night sky that was now associated with safety. This indicates that, although the participants showed a general disposition to prefer the day over the night sky, undergoing aversive apprehension associated with the day sky readily induced a reversal of this pattern.

Finally, future research is needed to overcome limiting aspects of the present study (e.g., trial number, sample size, and group composition) in order to clarify the role of interindividual differences in navigational performance (e.g., with respect to anxiety or aging; Wolbers and Hegarty, 2010; Lester et al., 2017; Goodman et al., 2020). While naturalistic tasks partly hinder experimental control, well-powered studies using virtual reality could address the tipping point between functional and dysfunctional effects of perceived environmental threat and safety. Here, a transdiagnostic approach may be of particular interest, for example, for large diverse samples ranging from low to high anxiety to anxious psychopathology (e.g., Robinson et al., 2013; Norton and Paulus, 2017).

## 5. Conclusion

The present study provides initial evidence for the view that the effect of aversive apprehensions on spatial navigation performance may depend on the nature of the navigation task. The performance in route retracing, an intuitive and often highly adaptive reaction, changes as a function of instructed threatening or safe environmental conditions, whereas the performance in route repetition does not. We also show that higher levels of trait anxiety improved the ability to retrace a route within a threatening context, whereas lower levels of trait anxiety were associated with worse performance under threat. Thus, besides its detrimental effects, trait anxiety may also support beneficial navigational performance, when it is congruent with adaptive avoidance behaviors.

## Data availability statement

Data and analyses scripts can be found in OSF using this link https://osf.io/3qdf9/.

## Ethics statement

The studies involving human participants were reviewed and approved by ethics committee (University of Heidelberg). The patients/participants provided their written informed consent to participate in this study.

## Author contributions

FB designed the study, supervised the data collection, contributed to the data analyses, wrote and revised the manuscript, and acquired the funding. PA programmed the experimental environment and revised the manuscript. MR designed the study, performed the data analyses, wrote and revised the manuscript, and acquired funding. All authors contributed to the article and approved the submitted version.

## Funding

This research was supported in part by the German Research Foundation (DFG) grants to FB (BU 3255/1-1 and -2) and MR (project number: 411006663). We also acknowledge the support by the Open Access Publication Fund of TU Berlin.

## Conflict of interest

The authors declare that the research was conducted in the absence of any commercial or financial relationships that could be construed as a potential conflict of interest.

## Publisher’s note

All claims expressed in this article are solely those of the authors and do not necessarily represent those of their affiliated organizations, or those of the publisher, the editors and the reviewers. Any product that may be evaluated in this article, or claim that may be made by its manufacturer, is not guaranteed or endorsed by the publisher.

## Footnotes

^1^Adding group as a separate factor in the model did not reveal significant results for the main effect of group or its interactions with other factors (all *p*s > 0.065).
